# The Dublin Meeting: The Menace of Trades Unionism

**Published:** 1919-03-22

**Authors:** 


### THE EDITOR'S LETTER-BOX.

**[Correspondence on all subjects is invited, but we cannot in any way be responsible for the opinions expressed by our correspondents, who must give their name and address as a guarantee of good faith, but not necessarily for publication. Correspondents are reminded that brevity of style and conciseness of statement greatly facilitate early insertion.]**

### THE DUBLIN MEETING: THE MENACE OF TRADES UNIONISM.

*To the Editor of* The Hospital.

Sir,—Judging from the tone of her letter, published in your issue of March 15, Miss Matheson, I regret to observe, would appear to be nettled. I still further regret that she is not more specific in making so grave an accusation against me as manufacturing "falsehoods," for, as I pointed out within the past week or two, general charges are singularly unsatisfactory. If there is a mis-statement or a falsehood, I like to see it set out between quotation marks.

We have two accounts of the same meeting, Miss Matheson's and mine. Miss Matheson challenges my story, attributes it to my imagination, and, in conclusion, affirms that truth is turned into falsehood. Pretty strong, eh? I can only guess that what is objected to is my statement that the meeting was successful—"appallingly successful." Even if my critic were correct I should have scarcely thought that the language was called for. However, this is a small matter, which I submit to the judgment of the reader.

There is no dispute as to the main facts. I accept Miss Matheson's description of the proceedings as entirely accurate. What have we then? (1) A crowded meeting assembled in trying and difficult circumstances, and when nurses' services were at a premium owing to the influenza epidemic. (2) Speeches by prominent trades unionists whose arguments Miss Matheson describes as "excellent," "no fault to find with," "particularly telling." (3) Miss Bennett, promoter and advocate of the Union, "undeniably disinterested and only willing to help." Voila!

I described the meeting as successful. From Miss Matheson's account how should you describe it? As regards the absence of a show of hands—on which, apparently, Miss Matheson builds her hopes and frames her accusation—what does it signify? In my esteem, nothing. Before the meeting forty of these women had enrolled as Union members, and at its conclusion thirty added their names. Up to the time of writing 150 have paid their entrance fees. Amongst the promoters the utmost activity prevails. On Saturday last a meeting for probationers was held. During this week three meetings are being held for midwives, Jubilee nurses, private nurses and nurses employed in private nursing homes. Special attention is being paid to Poor Law nurses, who are displaying much interest in the new movement. And, please note, the feature of the Union which is most attractive to recruits is a rule to the effect that hospital matrons are permitted to join only as ordinary members, and will not be permitted to act on the Executive unless they are duly elected by their fellow-members. These are the facts up to date. If I lied in describing this first venture of Trade Unionism into the realm of the nurse as "appallingly successful" I hope the reader will not pass judgment on me in wrath. Put it down rather to my clouded vision. One other matter, and I pass on. In my account of the meeting I omitted to refer to the "loudly applauded College member who objected to handing over her personal responsibility towards her patients to a committee of nurses or any one else." I quote from Miss Matheson's letter. I deliberately refrained from chronicling this speech, which was made by Miss Matheson herself, and for her own sake. It did not help the cause, but, on the contrary, evoked much unfavourable comment, not because of the sentiment, which is excellent, but because the speaker is not engaged as a nurse, and therefore has no patients to hand over. Moreover, so I am informed, Miss Matheson has never at any time had experience of Irish hospital nursing.

Personally, I regard the Trades Union in the nursing world as something to cause real alarm, and I think that the first step towards progress is to recognise rather than ignore the menace. To get angry because it is described as strong is pusillanimous. My reasons for objecting to a Trades Union are twofold.

I consider, in the first place, that such an alliance as that which it involves lowers the status of a body of trained women aiming after professional recognition. If it has to be done in order to obtain the redress of serious and substantial grievances, let it be a last resort. The time is not yet. The British hospital nurse is the employee, not of the Hospital Committee, but of the nation, and I now repeat, what I have already stated more than once, that her claims have never yet been presented to her employer. When the nation refuses to reward the nurse suitably for her labour, then by all means the Trades Union or any other union. In the meantime, it would be a fatal mistake for nurses trailing clouds of glory from the battlefield to lower the standard.

Apart from the alliance of the nurse with all sorts and conditions of skilled and unskilled labourers, I have a strong personal objection to the methods of a Trade Union. I refer to the forfeiture of my own identity, the surrender of my will and my judgment, and my promised acquiescence in any action that may be decided for me by others. A Trade Union is a tyranny which thousands of its own members regard with fear. I admit that it has proved a successful remedy against other tyrannies, but it is a desperate remedy, and should not be resorted to until all other means are tried and found wanting.

The dignified way is via the College. The College possesses potentialities to lead the nurse to the higher planes. The Trade Union means bondage and the lower levels. But the College must gird itself for conflict if the menace of Trades Unionism is to be overcome. The hope of the nurse is in the College militant.—Yours faithfully,

Ierne.

### IS NURSING A PROFESSION: STUDENTS OR PROBATIONERS?

*To the Editor of* The Hospital.

